# Comparison of three classification systems of Prepregnancy Body Mass Index with Perinatal Outcomes in Japanese Obese Pregnant Women: A retrospective study at a single center

**DOI:** 10.7150/ijms.47076

**Published:** 2020-07-25

**Authors:** Ryo Sugimura, Yukiko Kohmura-Kobayashi, Megumi Narumi, Naomi Furuta-Isomura, Tomoaki Oda, Naoaki Tamura, Toshiyuki Uchida, Kazunao Suzuki, Motoi Sugimura, Naohiro Kanayama, Hiroaki Itoh

**Affiliations:** 1 Department of Obstetrics and Gynecology, Hamamatsu University School of Medicine, Hamamatsu, Shizuoka, Japan.; 2 Department of Obstetrics, Gynecology and Family Medicine, Hamamatsu University School of Medicine, Hamamatsu, Shizuoka, Japan.

**Keywords:** prepregnancy, BMI, obese, classification, perinatal outcomes

## Abstract

In Japan, pregnant women are diagnosed as obese if the prepregnancy body mass index (BMI) is ≥25 kg/m^2^. However, this is different from other countries. The Institute of Medicine (IOM) classifies prepregnancy BMI as underweight (BMI <18.5 kg/m^2^), normal weight (BMI 18.5-24.9 kg/m^2^), overweight (BMI 25.0-29.9 kg/m^2^), and obese (BMI ≥30 kg/m^2^). In addition to these four categories, the American College of Obstetricians and Gynecologists (ACOG) classifies prepregnancy BMI as obesity class I (BMI 30.0-34.9 kg/m^2^), obesity class II (BMI 35.0-39.9 kg/m^2^), and obesity class III (BMI ≥40 kg/m^2^). We conducted a retrospective cohort study to compare obstetric outcomes by the three different categorizations in 6,066 pregnant women who gave birth between 2010 and 2019. According to Japanese classification, 668 (11%) pregnant women were classified as obese, and significant odds ratios (OR) were observed for hypertensive disorders of pregnancy (HDP; 3.32), gestational diabetes mellitus (GDM; 3.39), large for gestational age (LGA; 2.91), and macrosomia (4.01). According to the classification of IOM, 474 (7.8%) and 194 (3.1%) were classified as overweight and obese pregnant women, respectively. Specifically, a high OR was observed in obese pregnant women for HDP (5.85) and GDM (5.0). ACOG classification categorized 474 (7.8%) pregnant women as overweight, 141 (2.3%) as obesity class I, 41 (0.6%) as obesity class II, and 12 (0.2%) as obesity class III. In obesity class III, a significantly high OR was observed for HDP (12.89), GDM (8.37), and LGA (5.74). The Japanese classification may be useful for low-risk pregnancies, whereas IOM classification may be applicable to identify high-risk pregnancies. ACOG criteria may be useful for step-wise assessments of HDP and GDM risks in Japanese pregnant women; however, the number of class II and III obese pregnant women was small.

## Introduction

Obese pregnant women are associated with a high incidence of adverse outcomes in both mothers and infants, and the risk increases with increasing obesity. The Japan Society for the study of obesity (JASSO) decided to clarify obesity as peoples with BMI ≥25 kg/m^2^ in Japan, where the prevalence and degree of obesity remains mild 1. This is because the incidence of obesity-associated complications, such as hypertension, hypercholesteremia, and glucose intolerance were significantly higher in the peoples with BMI ≥25 kg/m^2^ than those with BMI <25 kg/m^2^ 1. The then Japanese Ministry of Health, Labour and Welfare defined pregnant women as obese if their prepregnancy body mass index (BMI) was ≥25 (kg/m^2^) in 2006 2. These official criteria are currently and widely used in Japan.

It is noted that this classification is different from those of Western countries. In the United States, the Institute of Medicine (IOM) has classified body weight based on prepregnancy BMI as underweight (BMI <18.5 kg/m^2^), normal weight (BMI 18.5-24.9 kg/m^2^), overweight (BMI 25.0-29.9 kg/m^2^), and obese (BMI ≥30 kg/m^2^) 3. On the other hand, the American College of Obstetricians and Gynecologists (ACOG) classifies pregnant women as underweight (BMI <18.5 kg/m^2^), normal weight (BMI 18.5-24.9 kg/m^2^), overweight (BMI 25.0-29.9 kg/m^2^), obesity class I (BMI 30.0-34.9 kg/m^2^), obesity class II (BMI 35.0-39.9 kg/m^2^), and obesity class III (BMI ≥40 kg/m^2^) 4, in consideration of the World Health Organization classification for men and nonpregnant women 5. Guidelines for Obstetrical Practice in Japan 2017 describe different recommendations by JASSO 6, the Japanese Ministry of Health, Labour and Welfare 2, and IOM 3 together, and state that there are different classifications in Japan 7.

Enomoto et al. assessed 97,157 women with singleton pregnancies registered in the Japan Society of Obstetrics and Gynecology (JSOG) Successive Pregnancy Birth Registry System, and compared Japanese and IOM classifications, demonstrating that BMI classification by the IOM is applicable to Japanese pregnant women 8. We recently suggested that the optimal gestational weight gain is slightly higher than that in the current recommendation in 101,336 women with singleton pregnancies using the same Registry System and 9. However, we cannot exclude the possibility that the heterogeneity of clinical practices in different institutions or hospitals, as well as diversity of regional lifestyles affect the pregnancy outcomes in the large-scale registry data analysis. Moreover, to the best of our knowledge, there have been few reports investigating the validity of the ACOG classification of prepregnancy BMI in Japanese pregnant women.

In the present study, we assumed that a retrospective analysis of pregnancy outcomes at a single center is based on consistent clinical practice and relatively similar reginal lifestyles of the subjects. Therefore, a single center study may support the findings obtained by analysis of large-scale registry data from across Japan.

We conducted a retrospective cohort study at a single center using the database to compare obstetric outcomes according to the three classifications, i.e., Japanese 2, 6, IOM 3, and ACOG 4.

## Methods

### Subjects

**Figure 1:**
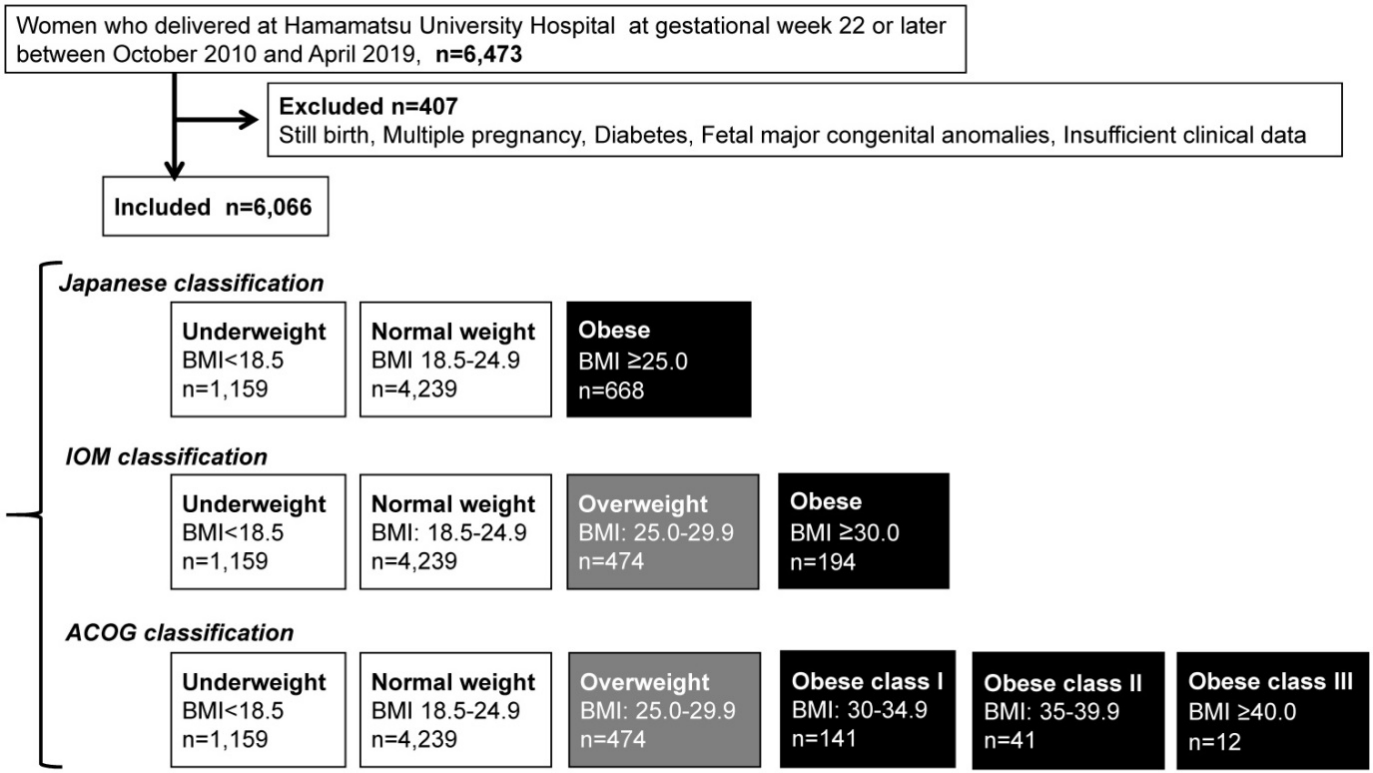
Flow diagram of study inclusion and three different classifications.

The present study was a retrospective investigation of women with singleton pregnancies delivered at Hamamatsu University Hospital at gestational week 22 or later. A total of 6,473 women were registered in the system between October 1, 2010 and April 30, 2019. The patient records were anonymized and deidentified prior to analysis. A total of 6,066 women were included in the study after exclusion of those with diabetes complications, history of delivering a newborn with congenital anomalies, or still birth, or whose data were insufficient (**Figure 1**).

### Selection and classification of obese pregnant women

We first selected obese pregnant women (n = 668) using the classification of the Japanese Ministry of Health, Labour and Welfare 2 and JASSO 6, i.e., prepregnancy BMI ≥25 kg/m^2^ (T**able 1A**) (**Figure 1**). Next, we compared their perinatal outcomes with those of pregnant women with normal weight (prepregnancy BMI 18.5-24.9 kg/m^2^, n = 4,239).

We subsequently selected overweight (n = 474) and obese pregnant women (n = 194) using the IOM classification (**Table 1B**) (**Figure 1**), and compared their perinatal outcomes with those of pregnant women with normal weight (n = 4,239).

Lastly, we selected overweight (n = 474), obesity class I (n = 141), obesity class II (n = 41), and obesity class III (n = 12) according to the ACOG classification (**Table 1C**) (**Figure 1**). Next, we compared their perinatal outcomes with those of pregnant women with normal weight (n = 4,239).

### Maternal backgrounds

The data of maternal age, primiparous rate, maternal height, prepregnancy BMI, maternal body weight gain, and gestational age at delivery were retrospectively collected from our clinical database. The data were separately assessed according to the three classifications of maternal obesity based on prepregnancy BMI.

### Perinatal outcomes

The following perinatal outcomes were assessed in the present study: hypertensive disorders of pregnancy (HDP), gestational diabetes mellitus (GDM), large for gestational age (LGA), small for gestational age (SGA), macrosomia, spontaneous preterm birth, post-term birth, postpartum hemorrhage (PPH) in cesarean section (CS), PPH in vaginal delivery (VD), neonatal asphyxia (Apgar score <7 points at 5 min after delivery), neonatal acidosis (umbilical arterial pH <7.20), and total cesarean delivery (both elective and emergency). HDP was defined as hypertension (systolic blood pressure ≥140 mmHg and/or diastolic blood pressure ≥90 mmHg) developing after 20 weeks of gestation 10. GDM (by 75-g oral glucose tolerance test) was diagnosed when at least one of the following was observed: fasting blood glucose level of ≥92 mg/dL, blood glucose level at 1 h of ≥180 mg/dL, or blood glucose level at 2 h ≥153 mg/dL. Macrosomia was defined as a neonatal birthweight ≥ 4,000 g. SGA was defined as a neonatal birthweight below the 10th percentile of the Japanese reference curves of birthweight for gestational week 11. LGA was defined as a neonatal birth weight above the 90th percentile of the Japanese reference curves of birth weight for gestational week 11. Induced preterm birth was defined as preterm delivery by CS or induction of labor due to obstetrical indications such as HDP or nonreassuring fetal status. Spontaneous preterm birth was defined as other than preterm birth medically indicated by cesarean section or labor induction. PPH in VD was defined as an estimated blood loss of 1,000 mL or more 12. PPH in CS was defined as an estimated blood loss of ≥2,000 mL 12. Post-term birth was defined as a delivery later than 42 weeks 0 days of gestation. Expected date of confinement was determined by the physician at the outpatient clinic based on the last menstrual period, ultrasonographic measurement of crown-rump length, estimated ovulation date, or date of embryonal transfer, if appropriate. If gestational age according to the last menstrual period differed by >7 days from that based on ultrasonographic measurement of crown-rump length at <11 weeks, the latter was used to assign a gestational age 13.

### Statistics

Data were expressed as means ± standard deviation. Belcurve for Excel Statistics software version (Social Survey Research Information Co., Ltd. Tokyo, Japan) was used for the statistical calculations. Statistical analyses included the one-way analysis of variance, followed by Tukey-Kramer and Bonferroni multiple comparisons to compare the maternal characteristics and pregnancy outcomes among the categories. A *p*-value of <0.05 was considered significant. Logistic regression analysis was used to estimate odds ratios (OR) and 95% confidence intervals (CI), adjusting for confounding variables, including maternal age, maternal body weight gain, and parity.

### Approval

The ethics committee of the Hamamatsu University School of Medicine approved all procedures (No. E19-203). Written informed consent was received after an explanation of the study.

## Results

### Classification of obese pregnant women using three criteria

According to the Japanese classification (prepregnancy BMI ≥25.0 kg/m^2^), 668 (11%) pregnant women were classified as obese (**Table 1A**) (**Figure 1**). According to the IOM, 474 (7.8%) and 194 (3.1%) pregnant women were overweight (prepregnancy BMI 25.0-29.9 kg/m^2^) and obese (prepregnancy BMI ≥30.0 kg/m^2^), respectively (**Table 1B**). According to the ACOG classification, 474 (7.8%), 141 (2.3%), 41 (0.6%), and 12 (0.2%) pregnant women were overweight, obesity class I, obesity class II, and obesity class III (**Table 1C**), respectively.

### Perinatal outcomes of obese pregnant women according to the Japanese classification

**Figure 2:**
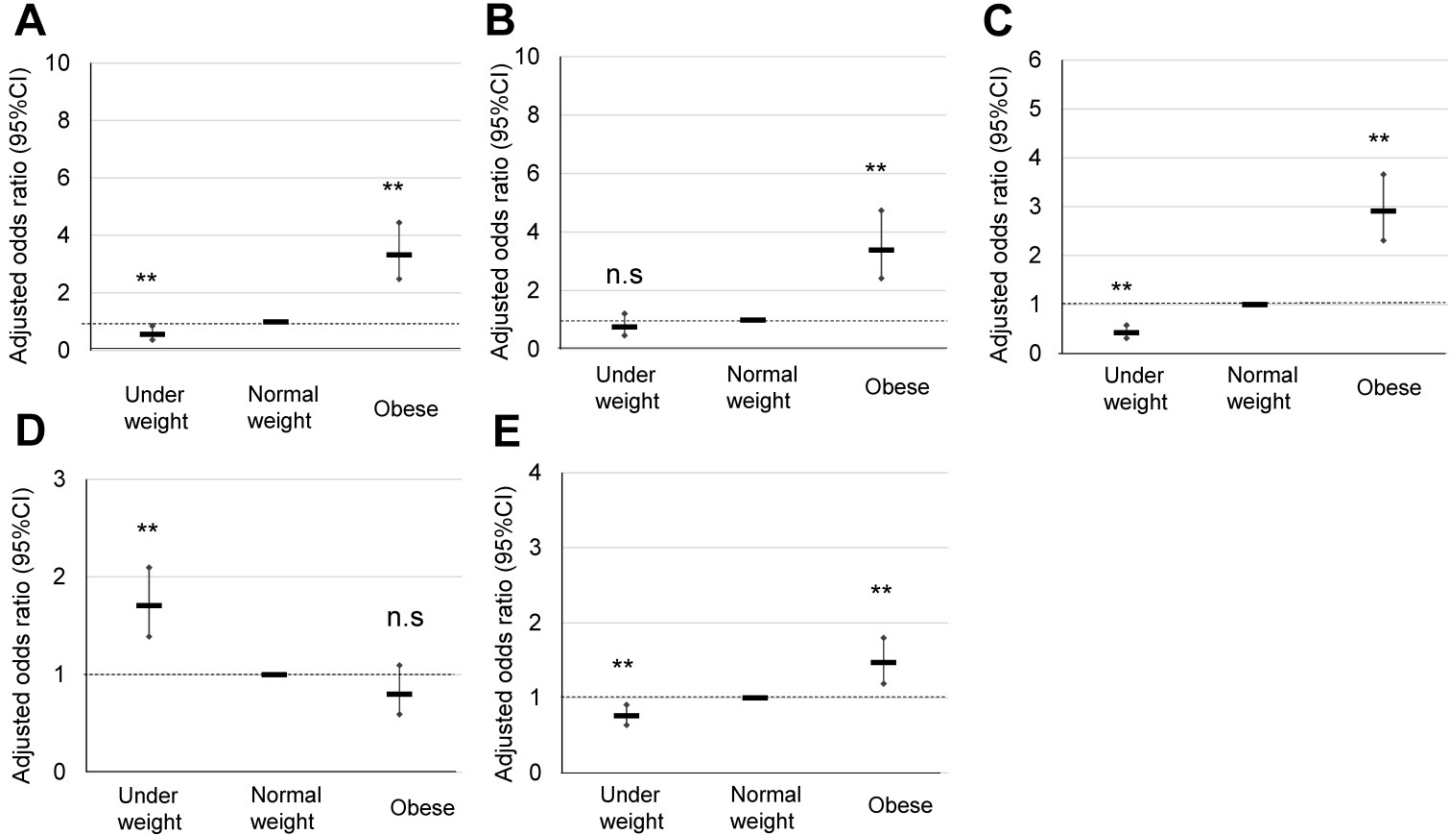
Comparison of pregnancy outcomes of obese pregnant women according to the Japanese classification**. A, B, C, D, and E** indicate HDP, GDM, LGA, SGA, and cesarean delivery rate, respectively, **; p <0.01.

The maternal characteristics of obese pregnant women according to the Japanese classification, and their comparison with those of normal and underweight pregnant women are summarized in **Table 1A**. Significant OR of HDP (3.32), GDM (3.39), LGA (2.91), and total CS were observed in the obese pregnant women (**Table 2A**) (**Figure 2**), for which explanatory variables were maternal age, primiparous rate, and maternal body weight gain (**Table 2B**).

### Perinatal outcomes of obese pregnant women according to the IOM classification

**Figure 3:**
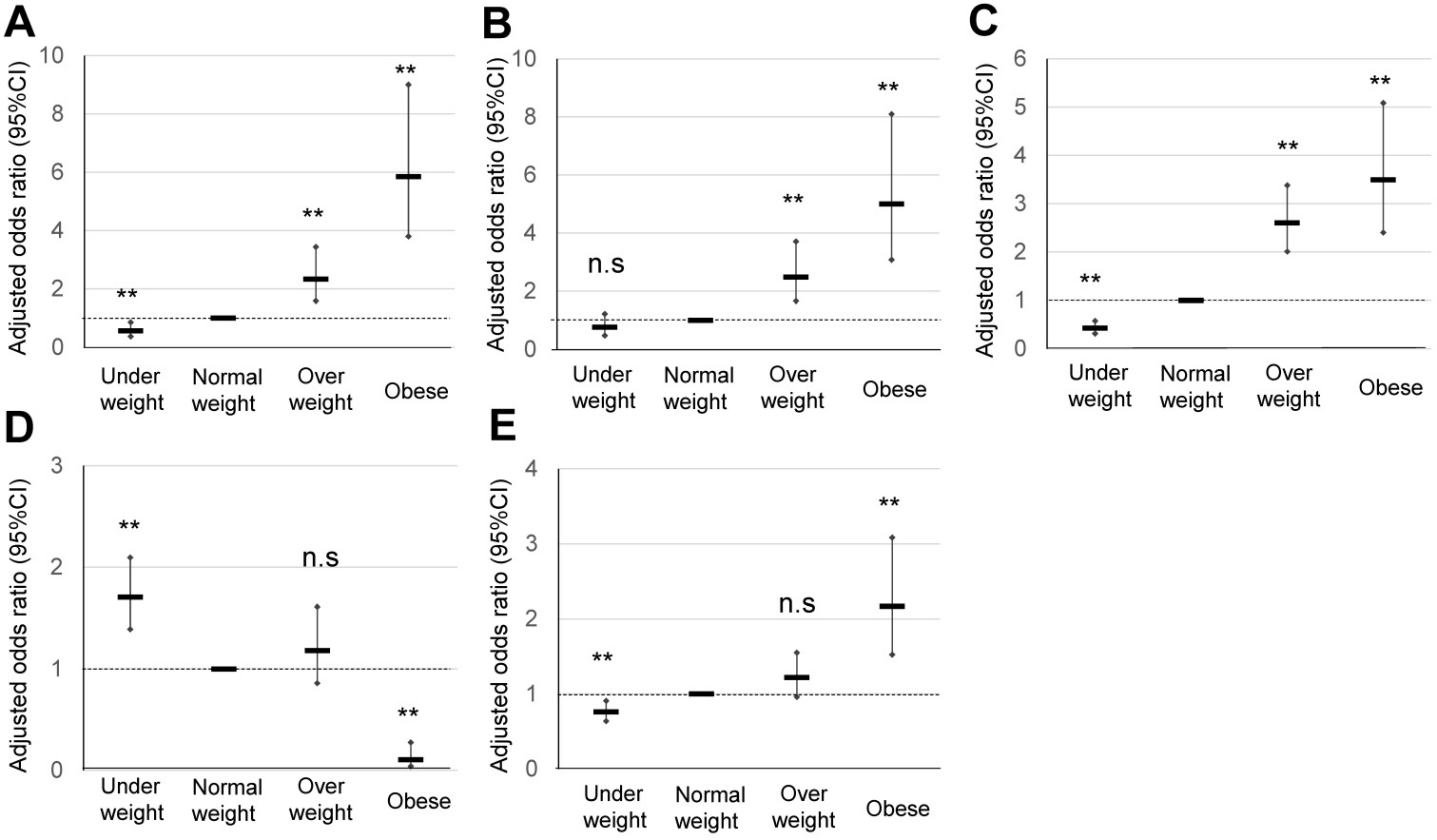
Comparison of pregnancy outcomes in obese pregnant women according to the IOM classification. **A, B, C, D, and E** indicate HDP, GDM, LGA, SGA, and cesarean delivery rate, respectively, **p <0.01.

The maternal characteristics of obese pregnant women according to the IOM classification, and their comparison with those of normal weight and overweight pregnant women are summarized in **Table 1B**. The significant OR of HDP in normal weight and overweight pregnant women was 1.23 and 5.85, respectively (**Table 3A**) (**Figure 3**). The significant OR of GDM in normal weight and overweight pregnant women was 2.48 and 5.00, respectively, whereas that of macrosomia in normal weight and overweight pregnant women was 2.86 and 6.95, respectively. A significant OR of PPH in CS (3.65), PPH in VD (1.76), and total CS (2.17) was observed in obese pregnant women, but not in overweight pregnant women. Explanatory variables of maternal age, primiparous rate, and maternal body weight gain are shown in **Table 3B**.

### Perinatal outcomes of obese pregnant women according to the ACOG classification

**Figure 4:**
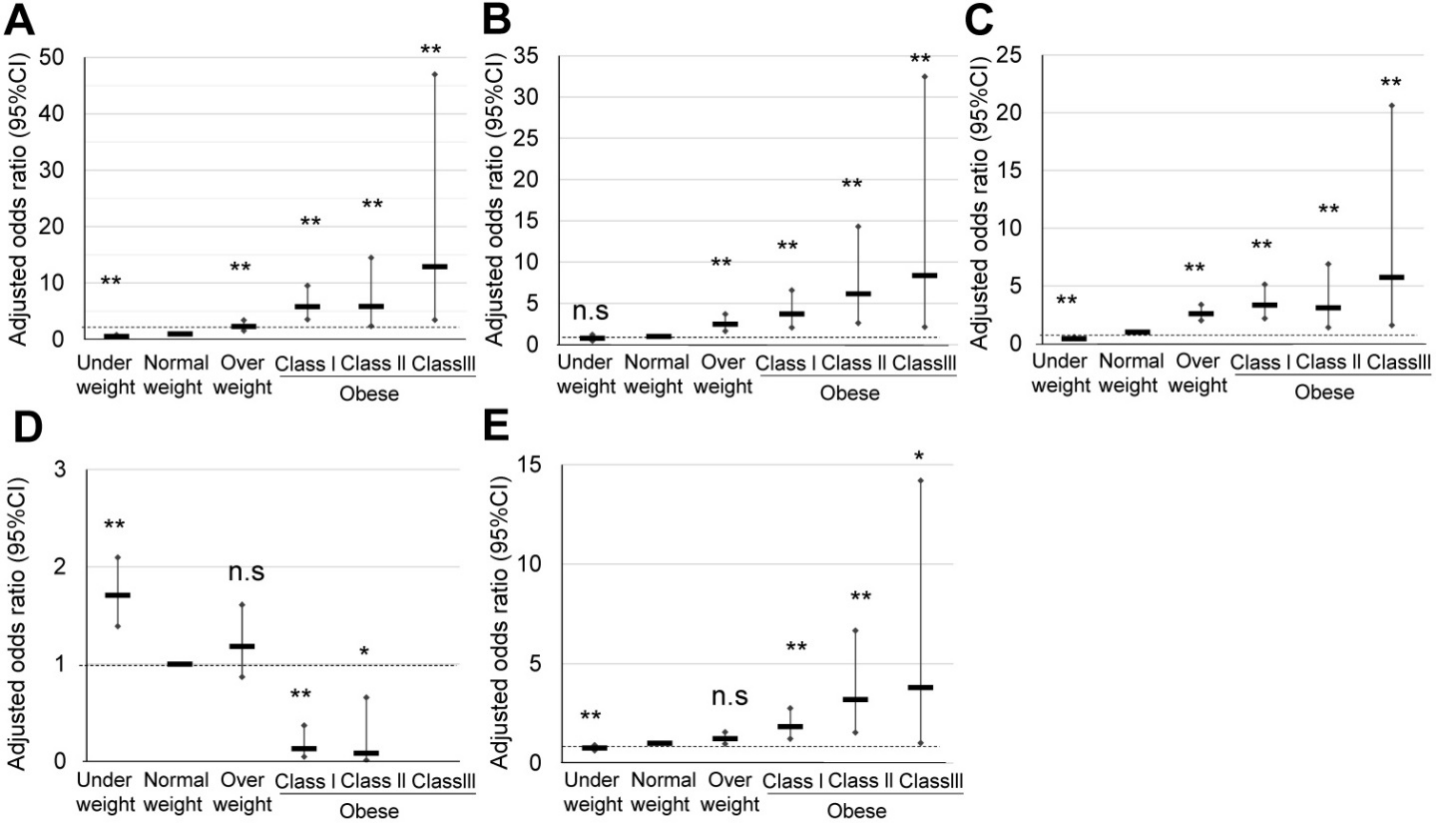
Comparison of pregnancy outcomes of obese pregnant women according to the ACOG classification. **A, B, C, D, and E** indicate HDP, GDM, LGA, SGA, and cesarean delivery rate, respectively, *; p <0.05, **; p <0.01.

The characteristics of maternal obesity class I, II, and III pregnant women defined according to the ACOG classification, and their comparison with those of normal weight and overweight pregnant women are summarized in **Table 1C**. In obesity class III pregnant women, a significantly high OR was observed in HDP (12.89), GDM (8.37), and LGA (5.74) (**Table 4A**) (**Figure 4**). However, the significant OR of HDP (5.91) and LGA (3.12) in the obesity class II pregnant women was similar to that observed for the obesity class I pregnant women, HDP (5.81), and LGA (3.35). Explanatory variables of maternal age, primiparous rate, and maternal body weight gain are shown in **Table 4B**.

## Discussion

In the present study, we simultaneously assessed pregnancy outcomes in obese pregnant women using the classification of the Japanese Ministry of Health, Labour and Welfare 2 and JASSO 6, i.e., prepregnancy BMI ≥25 kg/m^2^. In total, 668 (11%) pregnant women were classified as obese, and a significant OR was observed for HDP (3.32), GDM (3.39), LGA (2.91), and macrosomia (4.01) (Table 2A) (**Figure 2**). This Japanese classification is usually used to make a recommendation of body weight gain during pregnancy 2, 6, 7; however, the contribution of weight gain in pregnancy as an explanatory valuable was low, although sometimes significant (**Table 2B**). Therefore, this Japanese classification may be effective to identify approximately 10% of Japanese obese pregnant women with an OR of around 3 for the risk of representative complications of obesity in pregnancy such as HDP, GDM, LGA, and macrosomia.

According to the IOM classification system, 474 (7.8%) and 194 (3.1%) pregnant women were classified as overweight (prepregnancy BMI 25.0-29.9 kg/m^2^) and obese (prepregnancy BMI ≥30 kg/m^2^), respectively (**Table 1B**) (**Figure 1**). In Japan, 3.4% of women with a BMI ≥30 kg/m^2^ were in their 30s 14, which are similar to the findings of this study. Specifically, a high OR was observed in obese pregnant women for HDP (5.85) and GDM (5.0). A significant, but moderately high, OR was observed in PPH for VD (1.76) and total CS (2.17) (**Table 3A**) (**Figure 3**). This IOM classification is usually used to make a recommendation of body weight gain in pregnancy in the USA 3; however, the contribution of weight gain in pregnancy as an explanatory valuable was low, although sometimes significant (**Table 3B**). Therefore, this IOM classification may be effective to identify approximately 3% of Japanese obese pregnant women with an OR of around 5 for risks of GDM and HDP.

We finally categorized 474 (7.8%) pregnant women as overweight (prepregnancy BMI 25.0-29.9 kg/m^2^), 141 (2.3%) as obesity class I (prepregnancy BMI 30.0-34.9 kg/m^2^), 41 (0.6%) as obesity class II (prepregnancy BMI 35.0-39.9 kg/m^2^; n = 41), and 12 (0.2%) as obesity class III (prepregnancy BMI ≥40 kg/m^2^) according to the classification of ACOG (Table 1C) (**Figure 1**). In obesity class III, a significant and specifically high OR was observed for HDP (12.89), GDM (8.37), and LGA (5.74) (**Table 4A**) (**Figure 4**); however, only 12 pregnant women were included among 6,066 (**Figure 1**). Step-wise increases in the OR for HDP, GDM, and LGA were noted. The contribution of weight gain in pregnancy as an explanatory valuable was low, although sometimes significant (**Table 4B**). As the rate of obese pregnant women in Japan is lower than that in Western countries 14, 15, the ACOG criteria are applicable to relatively limited numbers of pregnant women in the Japanese society.

Prepregnant obesity is highly associated with perinatal complications. In the present study, three different prepregnancy BMI classification systems demonstrated marked differences in the pregnancy outcome profile among categorized groups of obesity. In this view, all three classification systems were applicable to Japanese pregnant women, although further assessment of obesity classes II and III is necessary because of the small numbers of subjects. As for the risk of SGA, the criteria of IOM and ACOG showed significant odds ratios of 0.11(IOM), 0.13 (ACOG; Class I), and 0.08 (ACOG; Class II), but no statistical significance by the criterion of JASSO (odds ratio of 0.81), which suggested possible better risk identification of the criteria of IOM and ACOG. On the other hand, all odds ratios of spontaneous preterm labor by the criteria of JASSO, IOM, and ACOG, were less than one and were of no statistical significance.

In Western countries, the population of obese pregnant women is higher than that in Japan, and there are many trials of interventions such as UK Pregnancy Better Eating and Activity Trial Intervention (UPBEAT) consortium 16, or Treatment of Obese Pregnant Women (TOP) study 17, suggesting that interventions for obese pregnant women are not always successful, especially in cases of extreme obesity like class II and III. Indeed, in the present study, in each of the three classification systems, the contribution of weight gain in pregnancy as an explanatory valuable was low. Therefore, it is important to use the prepregnancy BMI categorization for risk assessment and/or expectation for perinatal complications in obese pregnant women.

The current Japanese classification may be useful for screening of around 10% of obese pregnant women who are expected to be at a risk of GDM and HDP with an OR of around 3 (**Table 2A**) (**Figure 2**). It may be useful for conventional screening among low-risk pregnant women. The obesity classification of IOM (prepregnancy BMI ≥30 kg/m^2^ can identify obese pregnant women with an expected risk of GDM and HDP with an OR of around 5, which will enable identification of more high-risk pregnancies (**Table 3A**) (**Figure 3**). On the other hand, overweight pregnant women according to the IOM classification (prepregnancy BMI 25.0-29.9 kg/m^2^) had a significant but low OR of 1.23 for the risk of HDP (**Table 3A**) (**Figure 3**), which may be useful to exclude the high-risk population of HDP.

Among the three classifications, the definition of underweight is the same (prepregnancy BMI <18.5 kg/m^2^). The theme of this study was comparison of three classification systems of prepregnancy body mass index with perinatal outcomes in Japanese obese pregnant women. In this study, the low risk of HDP, GDM, LGA, and cesarean delivery, and high risk of SGA observed in underweight pregnant women were the same as in past reports 8, 18. Therefore in this study, we did not examine underweight pregnant women in detail.

The present study has some limitations. First, we carried out a retrospective analysis of pregnancy outcomes at a single center, which may not exactly represent the entire population of Japanese pregnant women. The incidences of Macrosomia and PPH were infrequent events in our study as other studies in Japan. In two major report based on JSOG nationwide database, the incidence of macrosomia were 0.76% 8 and 0.77% 9, approximately similar to the results of our observation (0.81%). Fukami reported that the incidence of PPH-VD was 8.7% at single tertiary perinatal medical center in Japan 19, which was roughly identical to that of our observation (11.5%). It is also noted that the current single center study is based on consistent clinical practice and relatively similar reginal lifestyles of the subjects. Therefore, it will reinforce the findings obtained by analysis of large-scale registry data across Japan 8, 9. Second, the data regarding the exact ethnicities of the subjects were not available. Third, we assessed the gestational weight gain as the change over the entire pregnancy period, and not as weekly weight gain. Fourth, dietary intervention was usually provided to obese pregnant women by dieticians, which may have affected the pregnancy outcome. Fifth the numbers of Class I, Class II Class III obesity by ACOG criteria were 141(2.3%), 41 (0.6%) and 12 (0.2%) and wide range of 95% CI were frequently observed in the analyses; therefore, more large-scale study is necessary to assess especially ACOG criteria.

In conclusion, the present data suggest that the two classification systems, the Japanese and IOM, are valid among Japanese pregnant women. However, the former is useful for screening obesity among low-risk pregnancies and the latter is applicable to identify high-risk pregnancies. The ACOG classification may be useful for step-wise assessments of the specific risks of HDP and GDM in Japanese pregnant women; however, further investigation is necessary because the numbers of class II and III obese pregnant women were small in the present study.

## Figures and Tables

**Table 1A:** Maternal characteristics of the study population according to the Japanese classification

	Underweight	Normal weight	Obese
Prepregnancy BMI (kg/m^2^)	<18.5; n = 1,159 (19.1%)	18.5 to 24.9; n = 4,239 (69.9%)	≥25; n = 668 (11%)
Maternal age (y)	30.8 ± 4.9**	31.9 ± 4.99	32.7 ± 4.6**
Primiparous rate (%)	57.1**	50.1	40.5**
Maternal height (cm)	158.2 ± 5.4	157.9 ± 5.5	157.3 ± 5.7
Total gestational weight gain (kg)	10.5 ± 4.0	10.7 ± 11.1	7.4 ± 6.1**
Gestational week at delivery	38.3 ± 2.1	38.4 ± 2.0	38.0 ± 2.6*

*p <0.05 vs Normal weight; **p <0.01 vs Normal weight.

**Table 1B:** Maternal characteristics of the study population according to the IOM classification

	Underweight	Normal weight	Overweight	Obese
Prepregnancy BMI (kg/m^2^)	<18.5; n = 1,159 (19.1%)	18.5 to 24.9; n = 4,239 (69.9%)	25 to 29.9; n = 474 (7.8%)	≥30; n = 194 (3.1%)
Maternal age (y)	30.8 ± 4.9**	31.9 ± 4.99	32.7 ± 5.1**	32.6 ± 4.6**
Primiparous rate (%)	57.1**	50.1	37.7**	47.4
Maternal height (cm)	158.2 ± 5.4	157.9 ± 5.5	157.4 ± 5.8	157.0 ± 5.4
Total gestational weight gain (kg)	10.5 ± 4.0	10.7 ± 11.1	8.3 ± 5.9**	5.3 ± 6.6**
Gestational week at delivery	38.3 ± 2.1	38.4 ± 2.0	38.1 ± 2.4*	37.7 ± 2.9**

*p <0.05 vs Normal weight; **p <0.01 vs Normal weight.

**Table 1C:** Maternal characteristics of the study population according to the ACOG classification

	Underweight	Normal weight	Overweight	Obese		
				Class I	Class II	Class III
Prepregnancy BMI (kg/m^2^)	<18.5	18.5 to 24.9	25 to 29.9	30 to 34.9	35 to 39.9	≥40
	n = 1,159 (19.1%)	n = 4,239 (69.9%)	n = 474 (7.8%)	n = 141 (2.3%)	n = 41 (0.6%)	n = 12 (0.2%)
Maternal age (y)	30.8 ± 4.9**	31.9 ± 4.99	32.7 ± 5.1**	32.7 ± 4.7	32.2 ± 4.1	32.4 ± 5.4
Primiparous rate (%)	57.1**	50.1	37.7**	46.1	51.2	50
Maternal height (cm)	158.2 ± 5.4	157.9 ± 5.5	157.4 ± 5.8	157.1 ± 5.1	157.3 ± 5.8	154.6 ± 7.4
Total gestational weight gain (kg)	10.5 ± 4.0	10.7 ± 11.1	8.3 ± 5.9**	6.5 ± 6.6**	1.8 ± 6.6**	2.8 ± 6.5*
Gestational week at delivery	38.3 ± 2.1	38.4 ± 2.0	38.1 ± 2.4*	37.7 ± 3.0**	37.8 ± 2.8	37.9 ± 2.3

*p <0.05 vs Normal weight; **p <0.01 vs Normal weight.

**Table 2A:** Comparison of pregnancy outcomes of obese pregnant women according to the Japanese classification

	Underweight	Normal weight	Obesity
Prepregnancy BMI (kg/m)	<18.5	18.5 to 24.9	≥25
	n = 1,159 (19.1%)	n = 4,239 (69.9%)	n = 668 (11%)
**HDP**			
% (n)	2.5% (n = 29)	4.0% (n = 169)	12.1% (n = 81)
Adjusted OR (95% CI)	0.57** (0.37-0.86)	1	3.32** (2.48-4.45)
**GDM**			
% (n)	1.9% (n = 22)	2.6% (n = 111)	11.2% (n = 75)
Adjusted OR (95% CI)	0.76 (0.48-1.22)	1	3.39** (2.42-4.74)
**LGA**			
% (n)	4.4% (n = 51)	9.5% (n = 402)	20.8% (n = 139)
Adjusted OR (95% CI)	0.43** (0.32-0.58)	1	2.91** (2.31-3.66)
**SGA**			
% (n)	13.5% (n = 156)	9.0% (n = 381)	4.6% (n = 31)
Adjusted OR (95% CI)	1.71** (1.39-2.10)	1	0.81 (0.59-1.09)
**Macrosomia**			
% (n)	0.2% (n = 2)	0.7% (n = 30)	2.5% (n = 17)
Adjusted OR (95% CI)	0.23* (0.05-0.96)	1	4.01** (2.18-7.37)
**Spontaneous preterm birth**			
% (n)	5.8% (n = 64)	2.5% (n = 100)	3.4% (n = 23)
Adjusted OR (95% CI)	2.33** (1.68-3.23)	1	0.81 (0.48-1.40)
**Post-term birth**			
% (n)	5.6% (n = 65)	8.0% (n = 339)	7.2% (n = 48)
Adjusted OR (95% CI)	0.62** (0.47-0.82)	1	1.01 (0.73-1.38)
**PPH (CS)**			
% (n)	1.7% (n = 4)	2% (n = 21)	0.7% (n = 5)
Adjusted OR (95% CI)	0.85 (0.29-2.50)	1	1.04 (0.37-2.91)
**PPH (VD)**			
% (n)	12.1% (n = 112)	15.8% (n = 503)	12.7% (n = 85)
Adjusted OR (95% CI)	0.80 (0.64-1.00)	1	1.45 (1.11-1.91)
**Neonatal acidosis**			
% (n)	7.1% (n = 81)	5.5% (n = 227)	5.1% (n = 34)
Adjusted OR (95% CI)	1.31* (1.01-1.72)	1	0.85 (0.57-1.27)
**Neonatal asphyxia**			
% (n)	2.8% (n = 32)	2.2% (n = 93)	3.0% (n = 20)
Adjusted OR (95% CI)	1.32 (0.88-2.00)	1	1.06 (0.47-2.39)
**Total CS**			
% (n)	20% (n = 229)	24.6% (n = 1,042)	34.6% (n = 231)
Adjusted OR (95% CI)	0.76** (0.64-0.91)	1	1.47** (1.19-1.80)

*p <0.05 vs Normal weight; **p <0.01 vs Normal weight.

**Table 2B:** Explanatory variables of HDP, GDM, LGA, SGA, and cesarean delivery in obese Japanese pregnant women according to the Japanese classification

Obesity comorbidity	OR	95% CI	*P*-value
**HDP**			
Maternal age (y)	1.05	1.03-1.08	<0.001
Primiparous rate (%)	0.47	0.36-0.61	<0.001
Body weight gain (kg)	1.00	1.00-1.01	0.516
**GDM**			
Maternal age (y)	1.09	1.06-1.13	<0.001
Primiparous rate (%)	0.95	0.70-1.29	0.740
Body weight gain (kg)	0.93	0.90-0.96	<0.001
**LGA**			
Maternal age (y)	1.02	1.00-1.04	0.042
Primiparous rate (%)	0.73	0.60-0.89	<0.001
Body weight gain (kg)	1.01	1.00-1.03	0.158
**SGA**			
Maternal age (y)	1.00	0.98-1.02	0.840
Primiparous rate (%)	1.06	0.87-1.30	0.552
Body weight gain (kg)	0.95	0.93-0.98	<0.001
**Cesarean delivery**			
Maternal age (y)	1.08	1.06-1.09	<0.001
Primiparous rate (%)	0.89	0.77-1.04	0.137
Body weight gain (kg)	1.01	1.00-1.02	0.197

**Table 3A:** Comparison of pregnancy outcomes of obese pregnant women according to the IOM classification

	Underweight	Normal weight	Overweight	Obesity
Prepregnancy BMI (kg/m)	<18.5	18.5 to 24.9	25 to 29.9	≥30
	n = 1,159 (19.1%)	n = 4,239 (69.9%)	n = 474 (7.8%)	n = 194 (3.1%)
**HDP**				
% (n)	2.5% (n = 29)	4.0% (n = 169)	8.4% (n = 40)	21.1% (n = 41)
Adjusted OR (95% CI)	0.57** (0.37-0.86)	1	1.23** (1.58-3.44)	5.85** (3.80-9.00)
**GDM**				
% (n)	1.9% (n = 22)	2.6% (n = 111)	8.5% (n = 40)	18% (n = 35)
Adjusted OR (95% CI)	0.76 (0.48-1.22)	1	2.48** (1.67-3.71)	5.00** (3.09-8.11)
**LGA**				
% (n)	4.4% (n = 51)	9.5% (n = 402)	19.7% (n = 93)	23.7% (n = 46)
Adjusted OR (95% CI)	0.43** (0.32-0.58)	1	2.61** (2.01-3.38)	3.50** (2.40-5.09)
**SGA**				
% (n)	13.5% (n = 156)	9.0% (n = 381)	5.5% (n = 26)	2.6% (n = 5)
Adjusted OR (95% CI)	1.71** (1.39-2.10)	1	1.18 (0.87-1.61)	0.11** (0.04-0.28)
**Macrosomia**				
% (n)	0.2% (n = 2)	0.7% (n = 30)	1.9% (n = 9)	4.1% (n = 8)
Adjusted OR (95% CI)	0.23* (0.05-0.96)	1	2.86** (1.33-6.15)	6.95** (3.11-15.53)
**Spontaneous preterm birth**				
% (n)	5.8% (n = 64)	2.5% (n = 100)	4.1% (n = 18)	2.6% (n = 5)
Adjusted OR (95% CI)	2.33** (1.68-3.23)	1	0.96 (0.54-1.70)	0.31 (0.10-0.96)
**Post-term birth**				
% (n)	5.6% (n = 65)	8.0% (n = 339)	7.6% (n = 36)	6.2% (n = 12)
Adjusted OR (95% CI)	0.62** (0.47-0.82)	1	0.94 (0.66-1.34)	0.44 (0.49-1.56)
**PPH (CS)**				
% (n)	1.7% (n = 4)	2% (n = 21)	0% (n = 0)	12.4% (n = 5)
Adjusted OR (95% CI)	0.85 (0.29-2.50)	1	-	3.65* (1.24-10.79)
**PPH (VD)**				
% (n)	12.1% (n = 112)	15.8% (n = 503)	18.9% (n = 61)	25.9% (n = 24)
Adjusted OR (95% CI)	0.80 (0.64-1.00)	1	1.35 (0.99-1.84)	1.76* (1.07-2.87)
**Neonatal acidosis**				
% (n)	7.1% (n = 81)	5.5% (n = 227)	5.9% (n = 27)	3.6% (n = 7)
Adjusted OR (95% CI)	1.31* (1.01-1.72)	1	0.98 (0.63-1.50)	0.57 (0.27-1.24)
**Neonatal asphyxia**				
% (n)	2.8% (n = 32)	2.2% (n = 93)	3.0% (n = 14)	3.1% (n = 6)
Adjusted OR (95% CI)	1.32 (0.88-2.00)	1	1.35 (0.76-2.38)	1.06 (0.47-2.39)
**Total CS**				
% (n)	20% (n = 229)	24.6% (n = 1042)	30.2% (n = 143)	45.3% (n = 88)
Adjusted OR (95% CI)	0.76** (0.64-0.91)	1	1.23 (0.97-1.56)	2.17** (1.52-3.08)

*p <0.05 vs Normal weight; **p <0.01 vs Normal weight.

**Table 3B:** Explanatory variables of HDP, GDM, LGA, SGA, and cesarean delivery in obese Japanese pregnant women according to the IOM classification

	OR	95% CI	*p* value
**HDP**			
***Overweight***			
Maternal age (y)	1.04	1.01-1.07	0.005
Primiparous rate (%)	0.40	0.29-0.54	<0.001
Body weight gain (kg)	1.00	1.00-1.01	0.191
***Obesity***			
Maternal age (y)	1.03	1.00-1.06	0.057
Primiparous rate (%)	0.41	0.30-0.57	<0.001
Body weight gain (kg)	1.00	1.00-1.01	0.153
**GDM**			
***Overweight***			
Maternal age (y)	1.01	1.05-1.13	<0.001
Primiparous rate (%)	1.13	0.81-1.59	0.474
Body weight gain (kg)	0.92	0.89-0.95	<0.001
***Obesity***			
Maternal age (y)	1.01	1.06-1.14	<0.001
Primiparous rate (%)	0.88	0.62-1.25	0.479
Body weight gain (kg)	0.92	0.89-0.95	<0.001
**LGA**			
***Overweight***			
Maternal age (y)	1.02	1.00-1.04	0.078
Primiparous rate (%)	0.70	0.58-0.86	<0.001
Body weight gain (kg)	1.01	1.00-1.02	0.201
***Obesity***			
Maternal age (y)	1.01	0.99-1.04	0.161
Primiparous rate (%)	0.78	0.64-0.96	0.020
Body weight gain (kg)	1.01	1.00-1.02	0.216
**SGA**			
***Overweight***			
Maternal age (y)	1.00	0.98-1.02	0.793
Primiparous rate (%)	1.07	0.87-1.32	0.494
Body weight gain (kg)	0.95	0.93-0.97	<0.001
***Obesity***			
Maternal age (y)	1.00	0.97-1.02	0.720
Primiparous rate (%)	1.08	0.86-1.34	0.512
Body weight gain (kg)	0.92	0.90-0.95	<0.001
**Cesarean delivery**			
***Overweight***			
Maternal age (y)	1.08	1.06-1.09	<0.001
Primiparous rate (%)	0.88	0.76-1.03	0.111
Body weight gain (kg)	1.01	0.99-1.03	0.196
***Obesity***			
Maternal age (y)	1.07	1.05-1.09	<0.001
Primiparous rate (%)	0.92	0.78-1.07	0.274
Body weight gain (kg)	1.01	0.99-1.02	0.335

**Table 4A:** Comparison of pregnancy outcomes of obese pregnant women according to the ACOG classification

	Underweight	Normal weight	Overweight	Obesity		
				Class I	Class II	Class III
Prepregnancy BMI (kg/m)	<18.5	18.5 to 24.9	25 to 29.9	30 to 34.9	35 to 39.9	≥40
	n = 1,159 (19.1%)	n = 4,239 (69.9%)	n = 474 (7.8%)	n = 141 (2.3%)	n = 41 (0.6%)	n = 12 (0.2%)
**HDP**						
% (n)	2.5% (n = 29)	4.0% (n = 169)	8.4% (n = 40)	20.6% (n = 29)	19.5% (n = 8)	33.3% (n = 4)
Adjusted OR (95% CI)	0.57** (0.37-0.86)	1	1.23** (1.58-3.44)	5.81** (3.58-9.52)	5.91** (2.41-14.5)	12.89** (3.53-47.03)
**GDM**						
% (n)	1.9% (n = 22)	2.6% (n = 111)	8.5% (n = 40)	13.5% (n = 19)	29.3% (n = 12)	33.3% (n = 4)
Adjusted OR (95% CI)	0.76 (0.48-1.22)	1	2.48** (1.67-3.71)	3.71** (2.09-6.58)	6.14** (2.64-14.3)	8.37** (2.16-32.48)
**LGA**						
% (n)	4.4% (n = 51)	9.5% (n = 402)	19.7% (n = 93)	23.4% (n = 33)	22.0% (n = 9)	33.3% (n = 4)
Adjusted OR (95% CI)	0.43** (0.32-0.58)	1	2.61** (2.01-3.38)	3.35** (2.19-5.12)	3.12** (1.41-6.88)	5.74** (1.60-20.6)
**SGA**						
% (n)	13.5% (n = 156)	9.0% (n = 381)	5.5% (n = 26)	2.8% (n = 4)	2.4% (n = 1)	0% (n = 0)
Adjusted OR (95% CI)	1.71** (1.39-2.10)	1	1.18 (0.87-1.61)	0.13** (0.05-0.37)	0.08* (0.01-0.65)	-
**Macrosomia**						
% (n)	0.2% (n = 2)	0.7% (n = 30)	1.9% (n = 9)	5.0% (n = 7)	2.4% (n = 1)	0% (n = 0)
Adjusted OR (95% CI)	0.23* (0.05-0.96)	1	2.86** (1.33-6.15)	8.91** (3.75-21.14)	3.03 (0.35-26.0)	-
**Spontaneous preterm birth**						
% (n)	5.8% (n = 64)	2.5% (n = 100)	4.1% (n = 18)	2.5% (n = 4)	2.8% (n = 1)	0% (n = 0)
Adjusted OR (95% CI)	2.33** (1.68-3.23)	1	0.96 (0.54-1.70)	0.329 (0.09-1.18)	0.147 (0.02-1.32)	-
**Post-term birth**						
% (n)	5.6% (n = 65)	8.0% (n = 339)	7.6% (n = 36)	6.4% (n = 9)	7.3% (n = 3)	0% (n = 0)
Adjusted OR (95% CI)	2.33** (1.68-3.23)	1	0.94 (0.66-1.34)	0.78 (0.39-1.55)	0.90 (0.28-2.94)	-
**PPH (CS)**						
% (n)	1.7% (n = 4)	2% (n = 21)	0% (n = 0)	6.7% (n = 4)	4.8% (n = 1)	0% (n = 0)
Adjusted OR (95% CI)	0.85 (0.29-2.50)	1	-	4.05* (1.21-13.62)	--	-
**PPH (VD)**						
% (n)	12.1% (n = 112)	15.8% (n = 503)	18.9% (n = 61)	25.9% (n = 21)	15% (n = 3)	0% (n = 0)
Adjusted OR (95% CI)	0.80 (0.64-1.00)	1	1.35 (0.99-1.84)	1.98* (1.16-3.36)	0.98 (0.26-3.65)	-
**Neonatal acidosis**						
% (n)	7.1% (n = 81)	5.5% (n = 227)	5.9% (n = 27)	4.4% (n = 6)	2.5% (n = 1)	0% (n = 0)
Adjusted OR (95% CI)	1.31* (1.01-1.72)	1	0.98 (0.63-1.50)	0.64 (0.27-1.51)	0.27 (0.04-2.10)	
**Neonatal asphyxia**						
% (n)	2.8% (n = 32)	2.2% (n = 93)	3.0% (n = 14)	2.8% (n = 4)	4.9% (n = 2)	0% (n = 0)
Adjusted OR (95% CI)	1.32 (0.88-2.00)	1	1.35 (0.76-2.38)	1.29 (0.47-3.55)	2.26 (0.54-9.50)	-
**Total CS**						
% (n)	20% (n = 229)	24.6% (n = 1,042)	30.2% (n = 143)	42.6% (n = 60)	20% (n = 21)	58% (n = 7)
Adjusted OR (95% CI)	0.76** (0.64-0.91)	1	1.23 (0.97-1.56)	1.84** (1.22-2.76)	3.20** (1.54-6.66)	3.80* (1.02-14.2)

**Table 4B:** Explanatory variables of HDP, GDM, LGA, SGA, and cesarean delivery in obese Japanese pregnant women according to the ACOG classification

	OR	95% CI	*p* value
**HDP**			
***Overweight***			
Maternal age (y)	1.04	1.01-1.07	0.005
Primiparous rate (%)	0.40	0.29-0.54	<0.001
Body weight gain (kg)	1.00	1.00-1.01	0.191
***Obesity class I***			
Maternal age (y)	1.04	1.01-1.07	0.020
Primiparous rate (%)	0.39	0.29-0.55	<0.001
Body weight gain (kg)	1.00	1.00-1.01	0.175
***Obesity class II***			
Maternal age (y)	1.03	1.00-1.06	0.056
Primiparous rate (%)	0.40	0.28-0.56	<0.001
Body weight gain (kg)	1.00	1.00-1.01	0.166
***Obesity class III***			
Maternal age (y)	1.04	1.01-1.07	0.020
Primiparous rate (%)	0.38	0.27-0.53	<0.001
Body weight gain (kg)	1.00	1.00-1.01	0.197
**GDM**			
***Overweight***			
Maternal age (y)	1.01	1.05-1.13	<0.001
Primiparous rate (%)	1.13	0.81-1.59	0.474
Body weight gain (kg)	0.92	0.89-0.95	<0.001
***Obesity class I***			
Maternal age (y)	1.09	1.05-1.14	<0.001
Primiparous rate (%)	0.94	0.66-1.35	0.738
Body weight gain (kg)	0.91	0.88-0.95	<0.001
***Obesity class II***			
Maternal age (y)	1.09	1.05-1.14	<0.001
Primiparous rate (%)	0.97	0.67-1.40	0.863
Body weight gain (kg)	0.90	0.87-0.94	<0.001
***Obesity class III***			
Maternal age (y)	1.09	1.05-1.14	<0.001
Primiparous rate (%)	1.07	0.73-1.58	0.717
Body weight gain (kg)	0.88	0.85-0.92	<0.001
**LGA**			
***Overweight***			
Maternal age (y)	1.02	1.00-1.04	0.078
Primiparous rate (%)	0.70	0.58-0.86	<0.001
Body weight gain (kg)	1.01	1.00-1.02	0.201
***Obesity class I***			
Maternal age (y)	1.01	0.99-1.03	0.252
Primiparous rate (%)	0.78	0.64-0.96	0.020
Body weight gain (kg)	1.01	1.00-1.02	0.207
***Obesity class II***			
Maternal age (y)	1.02	0.99-1.04	0.154
Primiparous rate (%)	0.76	0.61-0.94	0.011
Body weight gain (kg)	1.01	1.00-1.01	0.177
***Obesity class III***			
Maternal age (y)	1.01	0.99-1.03	0.288
Primiparous rate (%)	0.75	0.61-0.93	0.009
Body weight gain (kg)	1.00	1.00-1.01	0.180
**SGA**			
***Overweight***			
Maternal age (y)	1.00	0.98-1.02	0.793
Primiparous rate (%)	1.07	0.87-1.32	0.494
Body weight gain (kg)	0.95	0.93-0.97	<0.001
***Obesity class I***			
Maternal age (y)	1.00	0.97-1.02	0.728
Primiparous rate (%)	1.08	0.87-1.35	0.477
Body weight gain (kg)	0.92	0.90-0.95	<0.001
***Obesity class II***			
Maternal age (y)	1.00	0.98-1.02	0.777
Primiparous rate (%)	1.09	0.88-1.37	0.428
Body weight gain (kg)	0.93	0.90-0.95	<0.001
***Obesity class III***			
Maternal age (y)	-	-	-
Primiparous rate (%)	-	-	-
Body weight gain (kg)	-	-	-
**Cesarean delivery**			
***Overweight***			
Maternal age (y)	1.08	1.06-1.09	<0.001
Primiparous rate (%)	0.88	0.76-1.03	0.111
Body weight gain (kg)	1.01	0.99-1.03	0.196
***Obesity class I***			
Maternal age (y)	1.07	1.05-1.09	<0.001
Primiparous rate (%)	0.92	0.78-1.07	0.289
Body weight gain (kg)	1.01	0.99-1.02	0.305
***Obesity class II***			
Maternal age (y)	1.07	1.05-1.09	<0.001
Primiparous rate (%)	0.91	0.77-1.06	0.224
Body weight gain (kg)	1.01	0.99-1.02	0.370
***Obesity class III***			
Maternal age (y)	1.07	1.05-1.09	<0.001
Primiparous rate (%)	0.90	0.76-1.05	0.189
Body weight gain (kg)	1.00	0.99-1.01	0.383
